# Enzyme-Assisted Extraction of Fish Oil from Whole Fish and by-Products of Baltic Herring (*Clupea harengus membras*)

**DOI:** 10.3390/foods10081811

**Published:** 2021-08-05

**Authors:** Ella Aitta, Alexis Marsol-Vall, Annelie Damerau, Baoru Yang

**Affiliations:** Food Chemistry and Food Development, Department of Life Technologies, University of Turku, 20014 Turku, Finland; ella.v.aitta@utu.fi (E.A.); alexis.marsol@unizar.es (A.M.-V.); annelie.damerau@utu.fi (A.D.)

**Keywords:** enzymatic hydrolysis, by-products, fish oil, green technologies

## Abstract

Baltic herring (*Clupea harengus membras*) is one of the most abundant commercially caught fish species from the Baltic Sea. Despite the high content of fat and omega-3 fatty acids, the consumption of Baltic herring has decreased dramatically over the last four decades, mostly due to the small sizes and difficulty in processing. At the same time there is an increasing global demand for fish and fish oil rich in omega-3 fatty acids. This study aimed to investigate enzyme-assisted oil extraction as an environmentally friendly process for valorizing the underutilized fish species and by-products to high quality fish oil for human consumption. Three different commercially available proteolytic enzymes (Alcalase^®^, Neutrase^®^ and Protamex^®^) and two treatment times (35 and 70 min) were investigated in the extraction of fish oil from whole fish and by-products from filleting of Baltic herring. The oil quality and stability were studied with peroxide- and *p*-anisidine value analyses, fatty acid analysis with GC-FID, and volatile compounds with HS-SPME-GC-MS. Overall, longer extraction times led to better oil yields but also increased oxidation of the oil. For whole fish, the highest oil yields were from the 70-min extractions with Neutrase and Protamex. Protamex extraction with 35 min resulted in the best fatty acid composition with the highest content of eicosapentaenoic acid (EPA; 20:5n-3) and docosahexaenoic acid (DHA; 22:6n-3) but also increased oxidation compared to treatment with other enzymes. For by-products, the highest oil yield was obtained from the 70-min extraction with Protamex without significant differences in EPA and DHA contents among the oils extracted with different enzymes. Oxidation was lowest in the oil produced with 35-min treatment using Neutrase and Protamex. This study showed the potential of using proteolytic enzymes in the extraction of crude oil from Baltic herring and its by-products. However, further research is needed to optimize enzymatic processing of Baltic herring and its by-products to improve yield and quality of crude oil.

## 1. Introduction

Baltic herring (*Clupea harengus membras*) belongs to the Clupeidae family which also include sardines, shads, hilsa and menhadens. Many of the fishes in this family are recognized as the most important food fishes in the world. Furthermore, Atlantic herring (*Clupea harengus harengus*), which belongs to herrings, is the world’s most abundant fish species. Baltic herring is a subspecies of Atlantic herring, and it is the most important fished species in Finland both in value and volume [1].

The consumption of Baltic herring in Finland has dropped from over 30 million kg in the beginning of the 1980s to 3.5–4 million kg in 2019. The total catch, however, is approx. 100 million kg annually. This amount comprises approx. 90% of the catch from the Finnish sea areas, but most of it is used as feed for animals and in the production of fish meal and fish oil for fish farms [1]. The low domestic consumption of Baltic herring can be linked to at least two main reasons. Firstly, the amounts of dioxins and polychlorinated biphenyls (PCBs) exceeded the maximum limits set by the EU for a long time, which led to national recommendations for maximum intake. However, the latest research shows that dioxins remain under the maximum limits (3.5 pg/g WHO-PCDD/F-TEQ [2]) for Baltic herring which is under 19 cm long [3]. The reason for the lowered amounts is due to legislative restrictions in the Baltic Sea region, which have significantly lowered the pollution of these substances into the Baltic Sea. Secondly, the Baltic herring is a small fish which makes filleting processes more difficult. Due to the small size, approx. only 10% of the fish is suitable for automated filleting processes [4]. Seasonal differences in catch are also seen as low accessibility of fresh fish for consumers. There is an urgent need to improve the processing and value-addition of Baltic herring, as the feed market for fur animals is decreasing and the global market for high-quality fish oil and a sustainable source of proteins is rapidly growing.

The fat content of Baltic herring varies between 4–11% depending on the season, and the oil is especially rich in polyunsaturated omega-3 (n-3) fatty acids (FAs). Monounsaturated FAs (MUFAs) and polyunsaturated FAs (PUFAs) comprise approx. 39% and 25–29% of the Baltic herring lipids, respectively [5]. Moreover, Baltic herring is abundant in n-3 FAs eicosapentaenoic (EPA; 20:5n-3) and docosahexaenoic acid (DHA; 22:6n-3), found in concentrations of around 3 and 4 mg/g fresh weight (fw), respectively [6]. EPA and DHA have many beneficial functions in the human body, such as in cardiovascular, brain, neuronal, retinal and immune functions [7,8]. Due to the rapidly increasing demand, the price of fish oil for human consumption has risen threefold since the beginning of the 21^st^ century [9]. Given the abundance of Baltic herring and its high content of EPA and DHA, valorization of the oil for human consumption is a path that should be explored.

Conventional methods for fish oil extraction, typically wet rendering, can include heating and the use of organic solvents in the process. The former induces oxidation and degradation of heat-labile substances, the latter poses a risk for health and the environment. Within the frame of a more sustainable production, energy saving and safer green methods have been investigated for the extraction of fish oils [10] and protein isolates [11]. Examples of these methods include supercritical fluid extraction using carbon dioxide (SFE-CO_2_) [11,12], subcritical water extraction [13], enzymatic hydrolysis [14], fermentation [15], pH-shift processing, as well as ultrasound and microwave-assisted extractions [16,17]. Typically, the extraction methods focus primarily on either the protein or oil fraction, whereas the other is considered a secondary product. However, some methods, such as enzymatic hydrolysis using specific enzymes, allow the simultaneous extraction of both fractions from, e.g., whole fish or fish by-products, such as skin, heads, fins and viscera [18]. For example, a two-stage processing using mild thermal treatment together with enzymatic treatment allows the separation of high-quality oil and protein fractions [19]. Additionally, pH-shift processing can be used to extract proteins while removing up to 50% of the lipids [20].

Although enzyme-assisted extraction has been investigated for extracting oil from other fish species, to the best knowledge of the authors, no previous research has been reported on whole fish and by-products of Baltic herring. In this paper, we compared different enzymatic treatments in the extraction of fish oil from whole fish and by-products (side streams from filleting) of Baltic herring. The study focused on three different commercial enzymes, which were used with different hydrolysis times. Additionally, solvent extraction was used as a reference method to assess the performance of the enzymatic extraction. The obtained crudes were characterized in terms of yield and various parameters as quality indicators, including primary (peroxide value, PV) and secondary oxidation products (*p*-anisidine value, AV), fatty acid composition, and profiles of relevant volatile compounds. Our research provides not only novel scientific findings, but also guidance for green processing of Baltic herring as the most abundant yet underutilized fish species in the Baltic Sea.

## 2. Materials and Methods

### 2.1. Raw Materials and Chemicals

The raw materials used in the study were whole Baltic herring and filleting by-products consisting of heads, fins, tails and viscera from Martin Kala Oy (Turku, Finland). The fish was caught in November 2019 and frozen at −20 °C until analysis. The fish for pre-testing was gutted and beheaded Baltic herring from Martin Kala Oy, caught in October 2018. The fish and fish parts were received fresh, within one to two days after fishing (and filleting) and frozen immediately upon arrival.

Proteolytic enzymes Alcalase^®^, Neutrase^®^ and Protamex^®^ were donated by Novozymes (Bagsvaerd, Denmark). Methanol for the solvent extraction was purchased from Honeywell Riedel-de Haën Co. (Seelze, Germany) and chloroform from Thermo Fisher Scientific Inc. (Waltham, MA, USA). The reagents used in PV analysis were starch “Stärke 33615” from Honeywell Riedel-de Haën Co. (Seelze, Germany), glacial acetic acid and potassium iodate from VWR Chemicals (Leuven, Belgium) and sodium thiosulfate (Na_2_S_2_O_3_) from J.T. Baker Chemicals N.V. (Deventer, Holland). Reagents for the AV were *p*-anisidine from Sigma-Aldrich Co. (St. Louis, MO, USA) and iso-octane from Merck KGaA (Darmstadt, Germany). Methyl acetate and sodium for fatty acid methylation were purchased from Sigma-Aldrich Co. (St. Louis, MO, USA). The n-Hexane used in fatty acid analysis with GC-FID was purchased from VWR Chemicals (Leuven, Belgium). The internal standards used in fatty acid analysis were heptadecanoic acid (TG C:17) and triheneicosanoin (TG C:19) from Larodan AB (Solna, Sweden). The external standards used in volatile analysis were a homologous series of *n*-alkane standards (C7-C30 Saturated Alkanes) from Supelco (Bellefonte, PA, USA), 1-penten-3-ol and (E)-2-pentenal from Fluka (Buchs, Switzerland), acetaldehyde, acetic acid, propanal, hexanal, heptanal, octanal, nonanal, 2-ethylfuran, 2-pentylfuran and (*E*,*E*)-2,4-hexadienal from Sigma-Aldrich Co., and butanal and (E)-2-hexenal from Acros Organics (Carlsbad, CA, USA).

### 2.2. Enzyme-Assisted Extraction

The raw material was defrosted at 4 °C for 18–24 h. For each treatment, 200 g of fish was chopped into approx. 2 cm cubes and placed in a 1 L volumetric bottle, followed by the addition of 200 mL of tap water. Each treatment was conducted in triplicate. One of the proteolytic enzymes Alcalase^®^, Neutrase^®^ or Protamex^®^ was added in a concentration of 0.4 g/100 g fish. The enzyme dosage was chosen between the recommended concentration by Novozymes (0.1–0.2%) and the dosage commonly used in earlier research, i.e., 0.5% *w*/*w* [18]. The samples were kept in a water bath at 55 °C for 35 or 70 min, after which the water was heated to 90 °C and held for 15 min to inactivate the enzymes. The processing temperatures were recommended by the enzyme producer. The recommended reaction time for fish hydrolysate was 25–45 min, and therefore, two hydrolysis time points were chosen: one within the recommended time (35 min) and one extended time (70 min). The samples were cooled in an ice bath for 1 h. The samples were centrifuged at 4500 rpm for 20 min while cooling was set to 4 °C (Avanti jxn-26, Beckman Coulter, Brea, CA, USA). The oil layers from each triplicate were pooled together to limit the number of samples for analyses. The combined oil sample was centrifuged again at 3000 rpm for 5 min (Eppendorf Centrifuge 5804, Eppendorf AG, Hamburg, Germany). The sample tubes were flushed with nitrogen gas and stored at −80 °C. The pre-testing was conducted with the same conditions as mentioned before, but only the extraction time of 35 min was used. For the pre-testing, each treatment was conducted in triplicate.

### 2.3. Solvent Extraction

Modified Bligh and Dyer extraction using chloroform and methanol as solvents was used as a reference extraction method [21]. The changes made to the original method were: centrifugation at 4500 rpm for 10 min, and the solvent layers were evaporated with a rotary evaporator.

### 2.4. Yield

The amounts of crude oil from each extraction were measured gravimetrically. The crude oil fractions from triplicates were pooled together, and the yield was calculated as percentage of the raw material used.

### 2.5. Peroxide Value

The peroxide values (PVs) of the oil samples were determined according to the AOCS Cd 8-53 method (AOCS, 2004) using acetic acid and chloroform. For each analysis, 2.0 g of oil was weighed. An amount of 0.01 N Na_2_S_2_O_3_ was used as the titrant because the PVs of the crude oils were expected to be over 12. The starch indicator (10 g/L) was freshly prepared. Each sample was analyzed in triplicate.

### 2.6. p-Anisidine Value

The *p*-anisidine values (AVs) were determined by the AOCS Cd 18-90 method (AOCS, 2004), using 0.5 g of the extracted oil for each analysis. A blank was prepared for each sample using analytical-grade iso-octane. The samples were read at 350 nm with a spectrophotometer. Each oil sample was analyzed in duplicate.

### 2.7. Fatty Acid Analysis

The fatty acid analysis was conducted from the oil samples after converting FAs to fatty acid methyl esters (FAMEs) using the sodium methoxide catalyzed method [22] with a slight modification: the reaction was stopped with glacial acetic acid instead of oxalic acid. FAMEs dissolved in hexane were analysed with a gas chromatograph (Shimadzu GC-2010 equipped with AOC-20i auto injector, flame ionization detector, Shimadzu corporation, Kyoto, Japan). The injection (0.5 µL) was operated in a splitless mode with a sampling time of 1 min. Helium was used as the carrier gas. The column was a DB-23 (60 m × 0.25 mm i.d., liquid film 0.25 μm, Agilent Technologies, J.W. Scientific Santa Clara, CA, USA). The following temperatures were used: inlet temperature 270 °C; oven temperature 130 °C for 1 min, followed by increases of 6.5 °C/min to 170 °C, 2.75 °C/min to 205 °C (held 18 min) and 30 °C/min to 230 °C (held 2 min); detector 280 °C. The enzymatically extracted samples included triheptadecanoin and the solvent extracted samples included triheneicosanoin as internal standards. FAs were identified using external standards Supelco 37 Component FAME mix (Supelco, St. Louis, MO, USA) and 68D and GLC-490 (Nu-Check-Prep, Elysian, MN, USA). Correction factors were calculated using the external standards. The quantification of the FAs was conducted based on the internal standard area, concentration and correction factors. Each sample was analyzed in triplicate.

### 2.8. Volatile Analysis

The volatile compounds of the extracted crude oils were analyzed with head-space solid-phase micro extraction combined with gas chromatography and mass spectrometry (HS-HPME-GC-MS). For the analysis, 0.2 g of each oil sample was diluted in 2 mL of hexane. A volume of 0.5 mL of the dilution was pipetted into a 10 mL SPME headspace vial and the solvent was evaporated under nitrogen gas. The volatile compounds were collected with a divinylbenzene/carboxen/polydimethylsiloxane (DVB/CAR/PDMS, 50/30 µm) fibre from Supelco at 45 °C for 30 min under agitation using a TriPlus RSH multipurpose autosampler (Thermo Scientific, Waltham, MA, USA). The GC–MS analyses were conducted using a Trace 1310 Gas Chromatograph (Thermo Scientific™) with an SPB^®^-624 Fused Silica Capillary Column 60 m × 0.25 mm × 1.4 µm (Merck KGaA, Darmstadt, Germany). The GC was coupled to an ISQ 7000 Single Quadrupole Mass Spectrometer (Thermo Scientific™). Each analysis was carried out as a triplicate.

The incubation and extraction times of the samples were 20 min and 30 min, respectively. The samples were agitated at 40 °C. The fiber was cleaned at 250 °C for 2 min prior to extraction and 5 min after extraction. The volatiles were desorbed from the GC injector port for 5 min at 240 °C (splitless mode). Helium was used as a carrier gas in the gas chromatography at a flow rate of 1.4 mL/min. The oven temperature was programmed to hold at 40 °C for 6 min, after which it was increased to 200 °C at a rate of 5 °C/min and held at 200 °C for 10 min. Mass spectra were recorded in electron-impact (EI) mode at 70 eV within the mass range *m/z* 40−300. Chromeleon 7.0 (Thermo Scientific™) was used to operate the system. The main volatile compounds formed as products of lipid oxidation were tentatively identified based on retention index (RI) calculated using a homologous series of *n*-alkane standards from Supelco and NIST MS Search library (version 2.3, National Institute of Standards and Technology, Gaithersburg, MD, USA).

### 2.9. Statistical Methods

The differences in PV and AV were compared with one-way analysis of variance (ANOVA) and Tukey’s test in SPSS (IBM SPSS Statistics, version 25.0.0.1, IBM, New York, NY, USA). The FA data were analyzed with two-way ANOVA to discriminate the effects of hydrolysis times and enzymes. Further, the FA compositions of different enzymatic treatments were compared to solvent-extracted oil with one-way ANOVA. Significant differences for one-way ANOVA are reported for *p* < 0.05 and significant differences for both *p* < 0.05 and *p* < 0.001 are reported. Principal Component Analysis (PCA) using the Unscrambler^®^ X version 10.4.1 (Camo Process AS, Oslo, Norway) was applied to peak area data to determine differences in volatile profiles and to average data of selected measured parameters to determine the overall correlation between treatments and materials. The data were mean-centered and weighed (1/sdev) for PCA using Unscrambler.

## 3. Results and Discussion

### 3.1. Yield

The lipid yields of solvent and enzymatically extracted oils from pre-testing with gutted and beheaded Baltic herring and actual study samples with whole fish and by-products are presented in Table 1. The Bligh and Dyer solvent extraction is assumed to result in the recovery of more than 95% of the total lipids present in the raw materials, and therefore the total lipid contents were estimated to be 9.0% and 9.5% for whole fish and by-products, respectively. The fat content of the fish caught in autumn 2018 was similar to the levels reported in the literature [23]. In contrast, the oil content of the fish caught in autumn 2019 was higher than those reported in most previous studies, especially considering the findings of Rajasilta et al. (2018), who reported a decrease in the oil content of Baltic herring from 5–6% to 1.5% from 1987–2014. However, the oil contents were comparable to the oil content of Baltic herring fillets produced from fish caught in autumn [5]. The fish used was also caught in autumn, when Baltic herring has a higher fat content compared to the spring season [23,24]. The lipid content is also significantly higher than 4.5 g/100 g fw extracted from a catch from October 2020, which was analyzed by the authors (data not published). There seems to be a high variation in fat content of Baltic herring not only between seasons, but also between years.

The oil recoveries achieved by enzymatic extractions ranged from 3.9 to 6.3 g/100 g and 4.6 to 6.1 g/100 g for whole fish and by-products, respectively. The corresponding recovery percentages ranged from 43–69% for whole fish and 48–65% for by-products. Based on our results (Table 1), a longer extraction time gives higher results with Neutrase and Protamex, but this is not so evident for Alcalase. The best results were achieved with Neutrase using an extraction time of 70 min for whole fish, and Protamex with an extraction time of 70 min for by-products. The oil recoveries were similar to the results from the pre-test, except for Neutrase with 35 min extraction, which gave significantly lower results in the pre-test than in the actual extraction trial. This may have been related to the differences in total lipid content between the two batches of fish used, although this effect was not seen in the extractions using other enzymes. According to previous research, oil recovery from enzymatic hydrolysis has been reported as 72% from salmon by-products using 5% Alcalase [25], 51% from Catfish using 0.91% Alcalase [26], 76% from head, and 76% from whole mackerel using 2% Alcalase [27]. Neutrase has also been investigated, resulting in a lower recovery of oil compared to corresponding extraction with Alcalase. For example, treatment with Neutrase resulted in 36% oil recovery from cod by-products when the raw material was mixed with water (1:1 *w*/*w*) prior to extraction but increased to 64% when the raw material was hydrolyzed without added water. The water addition often enhances formation of an emulsion layer, which trapped some of the lipids [28]. In comparison, the best oil recovery reported in our study was 69% using 0.4% Neutrase, which is a smaller enzyme concentration than used in the studies mentioned above.

### 3.2. Fatty Acid Composition

The FA composition of different enzymatically extracted oils and solvent-extracted oil from whole fish are presented in Table 2. The FA composition of the oils extracted from by-products are presented in Table 3. Differences between the different enzymes and hydrolysis times were studied with two-way ANOVA, whereas the differences between different enzymatic treatments and solvent extraction were studied with one-way ANOVA.

The amount of saturated fatty acids (SFAs) was 26% of total fatty acids, MUFAs 38%, and PUFAs 36%. Further, the contents of n-3 and n-6 PUFAs were 28% and 8%, respectively. The lipid composition of whole Baltic herring is similar to those reported in the literature, where the proportions of n-3 PUFAs ranged from 25 to 28% and n-6 PUFAs from 8 to 9% [5]. Enzymatic treatments resulted in high amounts of n-3 and n-6 PUFAs. A 35-min extraction with Alcalase and a 70-min extraction with Neutrase yielded statistically lower content of PUFAs compared to other treatments. Together with a 35-min extraction with Protamex, these treatments led to lower n-3 PUFA contents compared to the rest, whereas the other enzymatic treatments resulted in a PUFA content similar to or better than the levels obtained with solvent extraction. The n-6 FAs were found in significantly higher concentrations in the enzymatically extracted oils than in the solvent-extracted oil.

The most dominant saturated fatty acid (SFA) was palmitic acid C16:0, which varied between 17.6 and 18.4% depending on the extraction. Further, the most dominant monounsaturated fatty acid (MUFA) was oleic acid C18:1 (n-9c), which ranged between 23.3 and 24.8%. These findings are in line with a previous study [29]. The most interesting findings concern the differences in PUFAs, the two n-3 FAs EPA (20:5n-3) and DHA (22:6n-3) being especially important. The EPA content in the oil from whole fish varied between 6.1 and 7.3%, the lowest values being from 70-min extractions with Neutrase and Protamex and the highest value from the 35-min extraction with Protamex. The tow-way ANOVA also shows that both the enzymes and hydrolysis times as well as their interaction (enzyme*time) had a significant impact on the n-3 PUFA content. The DHA content was significantly higher in the solvent-extracted oil compared to the enzymatically extracted oils, being 11.5% compared to 9.4–10.8%. In another study, DHA was the most abundant PUFA in Baltic herring oil (20.2 ± 4.9%) [29], whereas, in this study, despite being the most abundant PUFA, it ranged only between 9.4–10.8%. However, The DHA content was comparable to Baltic herring fillets (7.8%) and gutted Baltic herring (9.4%) [30]. The enzymatically extracted oils had DHA contents of 9.4–10.8%, the lowest values being obtained from the 35-min extractions with Neutrase and Alcalase, as well as the 70-min extraction with Protamex. The highest value was achieved with the 70-min extraction with Alcalase and the 35-min extraction with Protamex. Again, the two-way ANOVA showed significant differences between the enzymes and hydrolysis times as well as the impact of their interaction. The differences in these compositions may be partly from oxidation processes during the extraction because oxidation causes degradation of long-chain PUFAs. Further, differences in composition of different lipid classes extracted (not studied here) could cause differences in fatty acid profiles as certain lipid classes can be richer in certain fatty acids and vice versa for others [31].

In contrast to whole fish, the EPA and DHA contents in the oils from the by-products were not significantly different between any of the treatment groups (Table 3). The most abundant FAs were palmitic acid from SFA (17.8–19.4% of total FAs), oleic acid from MUFAs (24.7–25.5%) and DHA from PUFAs (8.3–9.3%). There are no significant differences in the compositions of SFAs, MUFAs, PUFAs or n-3 PUFAs between the treatment groups. In contrary, statistical differences (*p* < 0.05) are seen in n-6 PUFAs according to one-way ANOVA (all samples) but the two-way ANOVA (only enzymatically extracted samples) does not show significant differences between the enzymes or hydrolysis times. The FAs contributing to the biggest differences between the different enzymatic extractions were myristoleic acid (14:1 (n-5)) and palmitoleic acid (16:1 (n-7)), which were significantly different between the enzymes and hydrolysis times.

According to the fatty acid composition, the n-6/n-3 ratio in Baltic herring oil is 3.6/1 for whole fish and 2.9/1 for by-products. It is generally accepted that humans have evolved on a diet with an n-6/n-3 ratio of ~1. However, the Western diet has changed drastically over the last 100 years, and nowadays people are getting more n-6 FAs from their diets, typically from vegetable oils. The n-6/n-3 ratio in the Western diet is approx. 15/1–16.7/1. Higher ratios of n-6/n-3 FAs in the diet have been linked to a myriad of health-related issues, such as cardiovascular disease, cancer and inflammatory and autoimmune diseases. Individuals with diets rich in fish or supplementation with DHA and EPA have been associated with reductions in cardiovascular diseases and related mortality compared to those who do not consume fish [32,33]. Based on the fatty acid composition, oils from Baltic herring fish and by-products can be considered as a good source of n-3 PUFAs for human consumption. It is important to optimize the enzymatic extraction processes to minimize the loss of n-3 PUFAs caused by oxidation.

### 3.3. Oxidation

The AVs, PVs and total oxidation products (TOTOX = 2 × PV + AV) of enzymatically extracted oils are presented in Table 4. There were differences in AVs and PVs between the treatments. In the extractions from whole fish, AV was the lowest for the 35-min extraction with Alcalase, whereas the highest result was for the 70-min extraction with Protamex. With Baltic herring by-products, the lowest AV was achieved with the 35-min extraction using Neutrase, whereas the 70-min extraction with Alcalase resulted in the highest value, the difference being statistically significant (*p* < 0.05).

A longer extraction time resulted in a higher PV in all except one treatment. In addition to enzymes and extraction times, there was evidently an influence by the raw materials on the PVs of the crude extracts. The difference can be explained by interference of endogenous enzymes in the proteolytic process. With whole fish, the lowest PV was seen in the 35-min extraction with Alcalase, whereas the highest result was from the 70-min extraction with Protamex. For by-products, the lowest PV was from the 35-min extraction with Neutrase, which also had the lowest AV. The highest PV, on the other hand, was in the 70-min Alcalase-extracted oil, which also had the highest AV.

According to the TOTOX values, the 35-min extraction with Alcalase was the best for whole fish in terms of oxidative stability. On the other hand, the 35-min extraction with Neutrase was the best for by-products. The Codex standard for refined fish oil states that the acceptable limits are PV ≤ 5 meq/kg, AV ≤ 20 and TOTOX ≤ 26 [34]. In relation to these limits, all oil samples had a compromised oxidative status. A typical oil extraction process uses antioxidants, such as α-tocopherol or synthetic antioxidants, as oxidation protectants which were not used in this study. Further, the oils were not refined. During the typically used chemical refining process, oxidation products are removed, which improves both AV and PV. Additionally, fish oil rich in EPA and DHA can lead to over-estimation of AV because the absorbance intensity is dependent of the unsaturation level of the aldehydes [35]. Recently, the suitability of PV and AV as methods to determine the oxidative status of fish oils is being reconsidered and alternatives have been discussed [30,34].

Many oxidative compounds are derived from the degradation of long-chain PUFAs [36]. The degradation can also be visible as decreased DHA and EPA contents. In this study, Neutrase- and Protamex-extracted oils from whole fish had lower (*p* < 0.05) DHA and EPA contents after the 70-min extraction compared to the 35-min extraction. On the contrary, Alcalase had the opposite effect for both n-3 PUFAs. When looking at the total PUFAs, Neutrase and Protamex show a decreasing trend with a longer extraction time, the difference being statistically significant for Neutrase but not Protamex. The PUFA content of the Alcalase-extracted oil increased between the time points (*p* < 0.05). These findings do not correlate with the changes in AVs and PVs. Alcalase shows a significant increase in both AV and PV, although the EPA, DHA and PUFA contents increased with extraction time. For oils extracted from by-products, an increased extraction time decreased DHA with all enzymes and EPA with Alcalase and Neutrase; however, the differences were not significant. Furthermore, PUFAs showed a decreasing trend with increasing extraction time with Alcalase and Neutrase, but again, the differences were not statistically significant. The AVs increased significantly with Alcalase- and Neutrase-extracted oil with longer extraction times, but there were no statistically significant differences in PVs.

### 3.4. Volatiles

**Figure 1:**
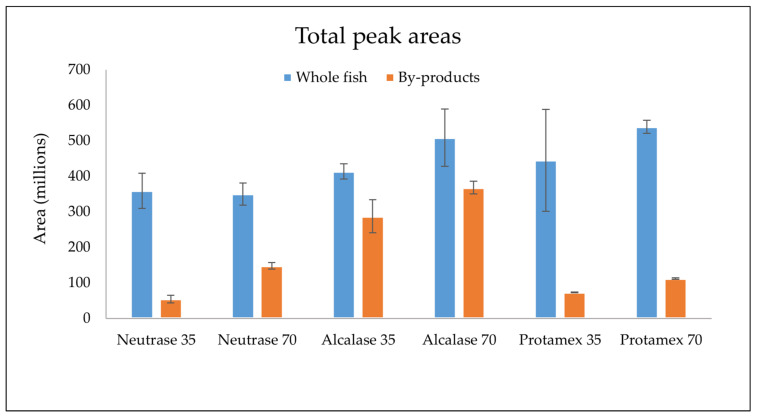
Total peak areas in HS-SPME-GC-MS chromatogram of all identified volatile compounds (N = 41) from different enzymatically extracted oils from whole fish (blue bars) and by-products (orange bars) of Baltic herring.

A total of 41 volatiles (Table 5) were tentatively identified from the oil samples based on the database NIST MS and comparison to external standards. Examples of total ion chromatograms for whole fish and by-products from the SPME-GC-MS are presented in Figure A3 and Figure A4, respectively. Retention indices (RIs) of the volatiles with retention times of 12.93 min or higher in Baltic herring crude oils were calculated based on the retention times of these compounds and *n*-alkane standards (Table 5). Most of the identified volatiles were secondary volatile compounds formed from lipid oxidation including propanal, butanal, 1-penten-3-ol, hexanal, heptanal and nonanal. A larger volatile content indicates a higher degree of lipid oxidation in fish oil, because the majority of the volatile compounds are oxidation products. The oils produced from whole fish had a larger total volatile content compared to those from by-products (Figure 1). Based on the total ion chromatogram of HS-SPME-GC-MS analysis, the most abundant volatiles in oils extracted from whole fish were 2-propenal, 2-ethylfuran, 1-penten-3-ol, hexanal and two unidentified compounds (unknown 1 and 2), whereas in the oils extracted from by-products, the most abundant volatile compounds were propanal, 2,3-butanedione, 2-ethylfuran, 1-penten-3-ol, 2,3-pentanedione, unknown 2, and (*E*,*E*)-2,4-heptadienal. Hexanal and 1-penten-3-ol are typical oxidation products from n-3 PUFA degradation and they are both related to unpleasant odors and flavors [37]. Both of these compounds have shown increases in Baltic herring during storage, indicating oxidation of long-chain unsaturated FAs [38]. (*E*,*E*)-2,4-heptadienal is another oxidation compound related to rancid and fishy odors. It has also been detected in oxidized herring oil and could be a good indicator for the oxidative status of fish oil [39]. Furthermore, 2-ethylfuran has been detected in fish, for example triploid rainbow trout, and it is derived from the oxidation of n-3 PUFAs. The odor of the compound is described as “rubber”, “pungent” and “green bean” [40,41].

**Figure 2:**
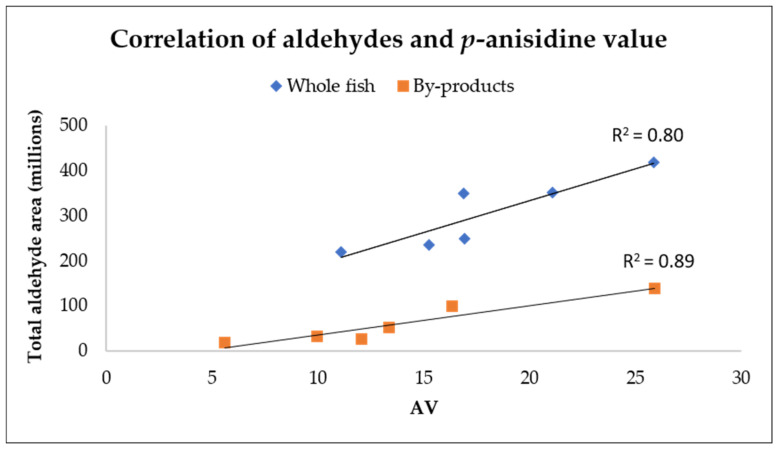
The correlation between *p*-anisidine value (AV) and total area of all identified aldehydes from the volatile analysis of enzymatically extracted oils from whole fish (blue) and by-products (orange) of Baltic herring.

Out of all the identified volatile compounds, 17 were aldehydes including acetaldehyde, 2-propenal, propanal, butanal, 2-methylbutanal, 3-methylbutanal, (*Z*)-2-butenal, (*E*)-2-pentenal, hexanal, 2-methyl-4-pentenal, (*E*)-2-hexenal, heptanal, (*E*,*E*)-2,4-hexadienal, (*Z*)-2-heptenal, octanal, (*E*,*Z*)-2,4-heptadienal and nonanal. Aldehydes are secondary oxidation products, which can also be measured with AV. The correlation between aldehydes and AV in the oils extracted from whole fish had a correlation factor of R^2^ = 0.80, whereas the corresponding one for by-products was R^2^ = 0.89 (Figure 2).

**Figure A1:**
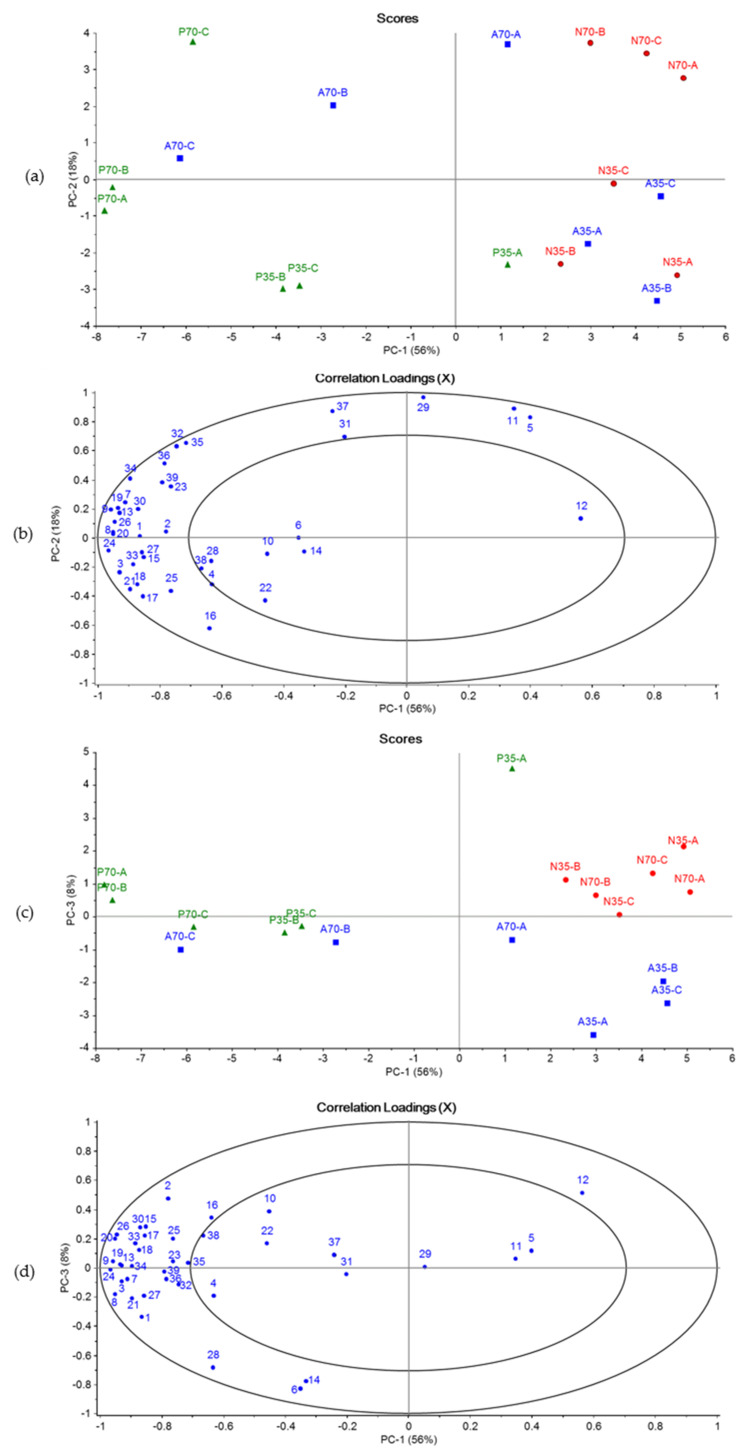
Principal component analysis (PCA) of volatile data from enzymatically extracted oils from whole Baltic herring. The samples are A: Alcalase (blue), N: Neutrase (red) and P: Protamex (green). The samples are also marked according to their extraction times of 35 or 70 min. (**a**) Scores plot of Principal Components (PC) 1 and 2; (**b**) correlation loadings of PC1 and PC2; (**c**) scores plot of PC1 and PC3 and (**d**) correlation loadings of PC1 and PC3. The numbered volatiles are (1) acetaldehyde, (2) 2-propenal, (3) propanal, (4) butanal, (5) 2,3-butanedione, (6) unknown 1, (7) formic acid, (8) 1,3-butanediol, (9) acetic acid, (10) 3-methylbutanal, (11) heptane, (12) 2-methylbutanal, (13) (*Z*)-2-butenal, (14) 2-ethylfuran, (15) 1-penten-3-one, (16) 1-penten-3-ol, (17) 2,3-pentanedione, (18) cyclopentanol, (19) propanoic acid, (20) (*E*)-2-pentenal, (21) hexanal, (22) 2-methyl-4-pentenal, (23) 3-hexanol, (24) 2,4-hexadien-1-ol, (25) (*E*)-2-hexenal, (26) heptanal, (27) (*E*,*E*)-2,4-hexadienal, (28) unknown 2, (29) 2-pentylfuran, (30) (*Z*)-2-heptenal, (31) trans-2-(2-pentenyl)furan, (32) octanal, (33) hexanoic acid, (34) (*E*,*Z*)-2,4-heptadienal, (35) (*E*,*E*)-2,4-heptadienal, (36) (*E*,*E*)-3,5-octadien-2-one, (37) 2-nonanone, (38) nonanal, (39) 5-ethyl-2(5H)-furanone, (40) (*E*,*E*)-3,5-Octadien-2-one and (41) nonanoic acid.

**Figure A2:**
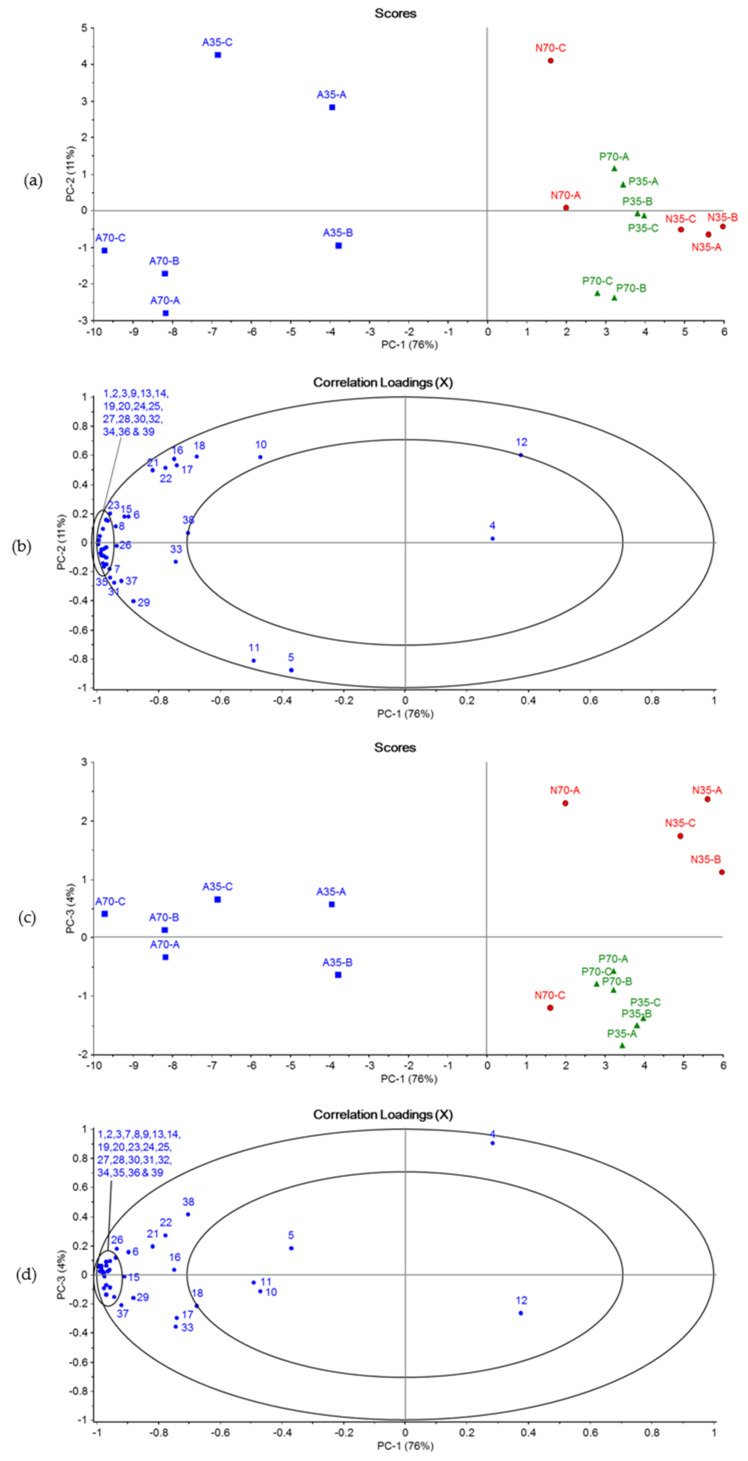
Principal component analysis (PCA) of volatile data from enzymatically extracted oils from by-products of Baltic herring. The samples are A: Alcalase (blue), N: Neutrase (red) and P: Protamex (green). The samples are also marked according to their extraction times 35 or 70 min. (**a**) Scores plot of Principal Components (PC) 1 and 2; (**b**) correlation loadings of PC1 and PC2; (**c**) scores plot of PC1 and PC3 and (**d**) correlation loadings of PC1 and PC3. The numbered volatiles are (1) acetaldehyde, (2) 2-propenal, (3) propanal, (4) butanal, (5) 2,3-butanedione, (6) unknown 1, (7) formic acid, (8) 1,3-butanediol, (9) acetic acid, (10) 3-methylbutanal, (11) heptane, (12) 2-methylbutanal, (13) (*Z*)-2-butenal, (14) 2-ethylfuran, (15) 1-penten-3-one, (16) 1-penten-3-ol, (17) 2,3-pentanedione, (18) cyclopentanol, (19) propanoic acid, (20) (*E*)-2-pentenal, (21) hexanal, (22) 2-methyl-4-pentenal, (23) 3-hexanol, (24) 2,4-hexadien-1-ol, (25) (*E*)-2-hexenal, (26) heptanal, (27) (*E*,*E*)-2,4-hexadienal, (28) unknown 2, (29) 2-pentylfuran, (30) (*Z*)-2-heptenal, (31) trans-2-(2-pentenyl)furan, (32) octanal, (33) hexanoic acid, (34) (*E*,*Z*)-2,4-heptadienal, (35) (*E*,*E*)-2,4-heptadienal, (36) (*E*,*E*)-3,5-octadien-2-one, (37) 2-nonanone, (38) nonanal, (39) 5-ethyl-2(5H)-furanone, (40) (*E*,*E*)-3,5-Octadien-2-one and (41) nonanoic acid.

**Figure A3:**
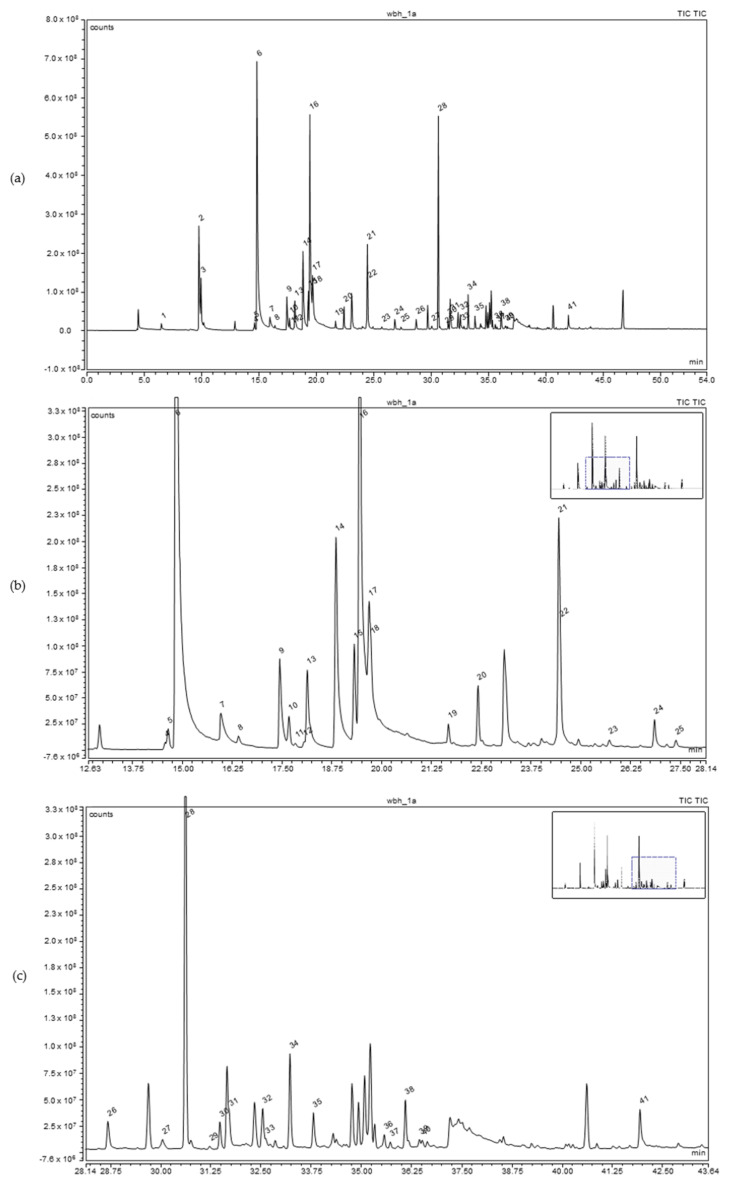
Total ion chromatogram of volatile analysis with HS-SPME-GC-MS from enzymatically extracted oil (Neutrase, 35 min) from whole Baltic herring. (**a**) Total ion chromatogram, (**b**) chromatogram scaled to retention time 12.63–28.14 min and (**c**) chromatogram scaled to retention time 28.14–43.64 min. The numbered volatiles are (1) acetaldehyde, (2) 2-propenal, (3) propanal, (4) butanal, (5) 2,3-butanedione, (6) unknown 1, (7) formic acid, (8) 1,3-butanediol, (9) acetic acid, (10) 3-methylbutanal, (11) heptane, (12) 2-methylbutanal, (13) (*Z*)-2-butenal, (14) 2-ethylfuran, (15) 1-penten-3-one, (16) 1-penten-3-ol, (17) 2,3-pentanedione, (18) cyclopentanol, (19) propanoic acid, (20) (*E*)-2-pentenal, (21) hexanal, (22) 2-methyl-4-pentenal, (23) 3-hexanol, (24) 2,4-hexadien-1-ol, (25) (*E*)-2-hexenal, (26) heptanal, (27) (*E*,*E*)-2,4-hexadienal, (28) unknown 2, (29) 2-pentylfuran, (30) (*Z*)-2-heptenal, (31) trans-2-(2-pentenyl)furan, (32) octanal, (33) hexanoic acid, (34) (*E*,*Z*)-2,4-heptadienal, (35) (*E*,*E*)-2,4-heptadienal, (36) (*E*,*E*)-3,5-octadien-2-one, (37) 2-nonanone, (38) nonanal, (39) 5-ethyl-2(5H)-furanone, (40) (*E*,*E*)-3,5-Octadien-2-one and (41) nonanoic acid.

**Figure A4:**
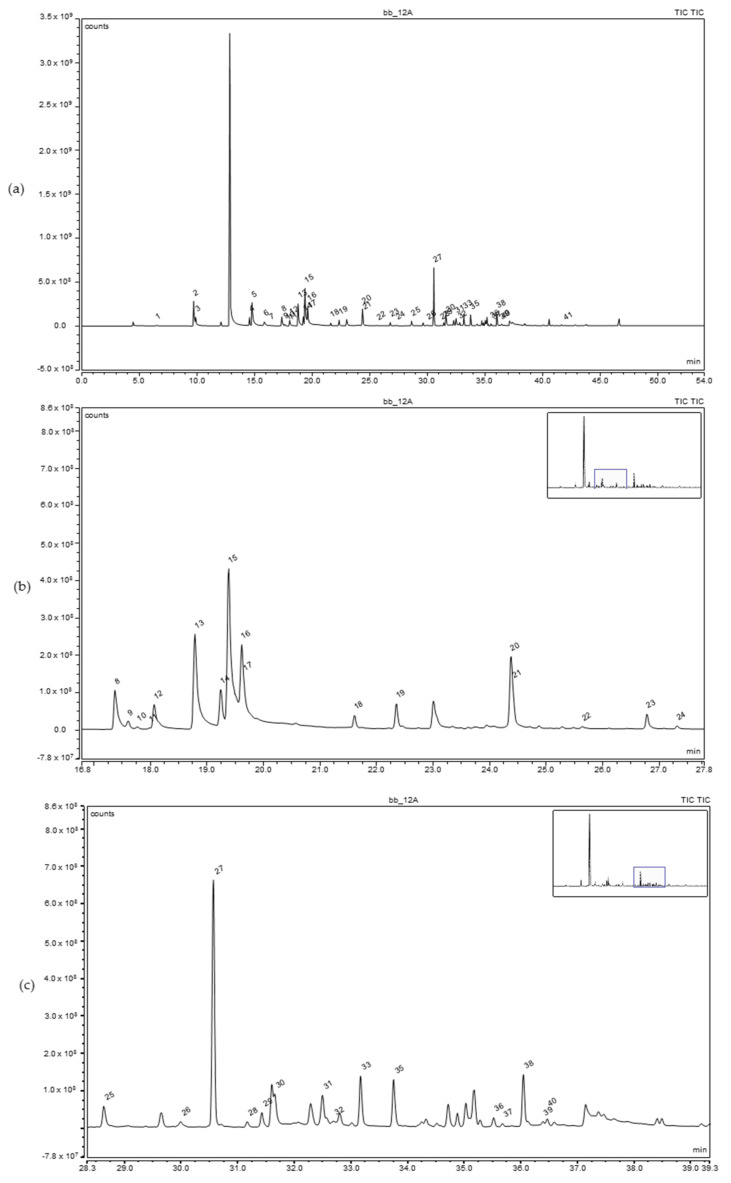
Total ion chromatogram of volatile analysis with HS-SPME-GC-MS from enzymatically extracted oil (Alcalase, 35 min) from by-products of Baltic herring. (**a**) Total ion chromatogram, (**b**) chromatogram scaled to retention time 16.8–27.8 min and (**c**) chromatogram scaled to retention time 28.3–39.3 min. The numbered volatiles are (1) acetaldehyde, (2) 2-propenal, (3) propanal, (4) butanal, (5) 2,3-butanedione, (6) unknown 1, (7) formic acid, (8) 1,3-butanediol, (9) acetic acid, (10) 3-methylbutanal, (11) heptane, (12) 2-methylbutanal, (13) (*Z*)-2-butenal, (14) 2-ethylfuran, (15) 1-penten-3-one, (16) 1-penten-3-ol, (17) 2,3-pentanedione, (18) cyclopentanol, (19) propanoic acid, (20) (*E*)-2-pentenal, (21) hexanal, (22) 2-methyl-4-pentenal, (23) 3-hexanol, (24) 2,4-hexadien-1-ol, (25) (*E*)-2-hexenal, (26) heptanal, (27) (*E*,*E*)-2,4-hexadienal, (28) unknown 2, (29) 2-pentylfuran, (30) (*Z*)-2-heptenal, (31) trans-2-(2-pentenyl)furan, (32) octanal, (33) hexanoic acid, (34) (*E*,*Z*)-2,4-heptadienal, (35) (*E*,*E*)-2,4-heptadienal, (36) (*E*,*E*)-3,5-octadien-2-one, (37) 2-nonanone, (38) nonanal, (39) 5-ethyl-2(5H)-furanone, (40) (*E*,*E*)-3,5-Octadien-2-one and (41) nonanoic acid.

The volatile data were analyzed with principal component analysis (PCA). The volatile data from whole fish (Figure A1) and by-products (Figure A2) were analyzed separately. The figures include scores and correlation loadings of the most important principal components (PCs). PCs 1 and 2 explain a total of 74% of the variance in the volatile data of oils extracted from whole fish. PC2 allows a quite good separation between faster extraction processes (35 min, lower scores on PC2) and slower ones (70 min, higher scores on PC2) independently to the enzyme used. The most influential compounds for PC2 are 2-pentylfuran, trans-2-(2-pentenyl)furan, 2-nonanone, 2,3-butanedione and heptane on the positive side of the X-axis, while 1-penten-3-ol was influential on the negative side. PC1 and PC3 (Figure A1c,d) explain 64% of the variance in the volatile data. PC3 explains only 8% of the variance but it allows a rather good separation of the Alcalase-extracted samples from those extracted with the other two enzymes. The correlation loadings show that most of the volatiles are grouped close to each other on the negative side of the Y-axis. The only volatile on the positive side of the Y-axis with a significant impact (inside the further ellipse) is 2-methylbutanal. The differences in total volatile peak areas (Figure 1) are also visible in the correlation loadings. The Neutrase-treated samples show a negative correlation with most volatiles, and these samples had the smallest volatile peak areas compared to the samples with other enzymes. The 70-min extractions using Alcalase and Protamex, on the other hand, correlate with most of the volatiles, indicating increased oxidation. 

The scores plot for PC1 and PC2 from oils extracted from by-products (Figure A2a) show that Alcalase samples separate well from Neutrase and Protamex samples. PC1 and PC2 explain 87% of the variance in the dataset. PC1 seems to relate to the differences between the enzymes, whereas PC2 show no clear trend in treatment time or other known factors. The correlation loadings (Figure A2b) show that most volatiles group on the negative side of the Y-axis, whereas 2-methylbutanal is the only volatile with a significant effect on the positive side. 2-Methylbutanal has a negative correlation with heptane and 2,3-butanedione. Butanal is also located on the positive side of the Y-axis, but it does not have a significant effect in the model with PC1 and PC2. The separation of Alcalase from Protamex and Neutrase can be explained by Figure 1 and Table 4, which show that Alcalase-treated samples are more oxidized and have bigger total volatile peak areas. Alcalase samples are also grouped closer to the large cluster of volatiles compared to Neutrase and Protamex samples. PC1 and PC3 (Figure A2c) group different enzymes from each other even clearer. PC3 explains 4% of the variance in the data set, but it allows a better separation of Neutrase and Protamex. According to the correlation loadings, butanal and 2-methylbutanal are the only compounds that have a significant effect on the positive side of the Y-axis, whereas other compounds are grouped together on the negative side. 

**Figure 3:**
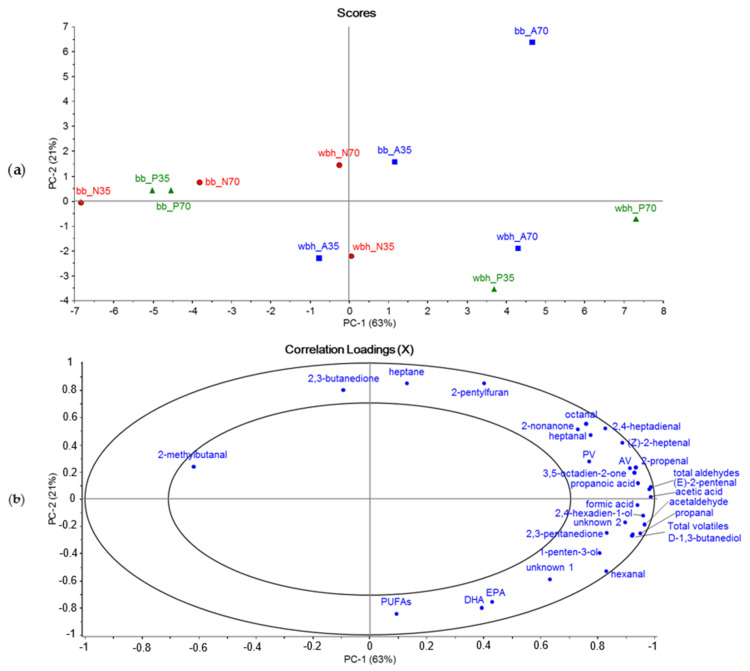
Principal component analysis (PCA) of volatile compounds that were most influential in PCAs with all identified volatiles, total volatile and aldehyde peak areas, EPA, DHA and PUFA contents, and AVs and PVs. Both raw materials were analyzed together and marked to the figures as bb (by-products) and wbh (whole Baltic herring). The enzymes are marked as their initials: A for Alcalase, N for Neutrase and P for Protamex. The figures are (**a**) scores plot and (**b**) correlation loadings of principal components PC1 and PC2. The data was normalized (auto normalization) and centered (mean centering).

The PCA models described above were used to select volatile compounds that contributed most to the first three principal components: formic acid, acetic acid, acetaldehyde, propanal, 2-propenal, propanoic acid, 2-methylbutanal, 2,3-butanedione, 1,3-butanediol, (*E*)-2-pentenal, 2,3-pentanedione, 1-penten-3-ol, 2-pentylfuran, hexanal, 2,4-hexadien-1-o, heptane, heptanal, (*Z*)-2-heptenal, 2,4-heptadienal, octanal, 3,5-octadien-2-one, 2-nonanone, unknown 1 and unknown 2. These compounds were important in oil samples from both raw materials. A new PCA model was created using these compounds, total volatile peak areas, total aldehyde areas, n-3 and n-6 PUFA contents, as well as PVs and AVs as variables. Figure 3 contains scores and correlation loadings where both raw materials are plotted together. PC1 and PC2 explain 84% of the variance in the data set. The oil samples extracted from whole fish (wbh) are grouped mostly on the negative side of the X-axis, while all by-product (bb) oils are on the positive side. Further, Alcalase-extracted oils from by-products clearly separate from Neutrase and Protamex samples along the X-axis. Based on the oxidation results discussed earlier, PC1 is related to oxidation because the most oxidized samples group on the right side of the X-axis, which correlate with AV, PV, aldehydes and most of the volatiles. On the other hand, 2-methylbutanal, which has been characterized with malty and dark chocolate odors [42], seems to be correlated with the less-oxidized samples (Neutrase- and Protamex-extracted oils from whole fish). PC2 explains 21% of the variance, and it allows moderate separation of the samples based on their extraction times. The compounds influential on the positive side of the X-axis are 2,3-butanedione, heptane and 2-pentylfuran. 2,3-Butanedione has been detected from other fish species, such as obscure puffer (*Takifugu obscurus*), and it has “sweet” and “caramel-like” odors [43]. 2-Pentylfuran has been reported to derive from the degradation of n-6 PUFAs and it has been detected in many fish species. It has been characterized with “orange” and “licorice” odors. [44,45]. On the negative side of the X-axis, however, the influential factors are EPA, DHA and PUFA contents. The FA analysis (Table 2) showed that the 35-min extraction with Protamex from whole fish lead to the highest DHA, EPA and PUFA contents, and the PCA models supports these findings. It can also be seen that the concentrations of these FAs are higher from the oils extracted from whole fish compared to by-products.

This study showed the potential of enzymatic hydrolysis for the extraction of oil from whole fish and by-products of Baltic herring. Our findings indicate that different enzymes and extraction times lead to a different oil composition and stability. One limitation of the current research is the lack of statistical comparison between different enzymes and hydrolysis times due to the pooling of the crudes from different extraction replicates. The pooling was performed to reduce the total number of samples for analysis. The oil recoveries achieved in this study would not be sufficient in the industry, where recovery rates of 70% or higher are considered optimal. The optimization of extraction parameters such as enzyme dosage was not performed to increase the oil yields in this study. A clear challenge for enzymatic hydrolysis is emulsion formation, which further reduces oil yield, as seen by the authors during hydrolyses of leaner Baltic herring fish from autumn 2020 (data not published). The interaction of oils and proteins, as well as endogenous enzymes in the raw material likely interfere with the processing. Further, the crude oil should be refined and enrichment with n-3 FAs could be investigated in order to obtain products with quality meeting the requirements set for fish oil for human consumption. These aspects will be the focus of our future research.

## 4. Conclusions

The best conditions for the enzymatic treatment were dependent on both the enzymes and the raw material. For whole fish, Neutrase led to the best yield, and the 70-min extraction time was better than 35 min. Neutrase also led to a smaller total volatile content than the other two enzymes; however, treatment with Alcalase gave the smallest TOTOX values. In contrast, extraction with Protamex resulted in the most oxidized oils. For by-products, Protamex with a 70-min extraction time led to the highest yield. Both hydrolysis times with Protamex resulted in oils with lower total volatile contents compared to treatment with Alcalase, and the longer extraction time with Neutrase. The PCA models show distinctive differences in the oils extracted with different enzymes. Neutrase and Protamex extractions showed a negative correlation with volatile oxidation products in the PCA model. Further research is needed to optimize the enzyme-assisted oil extraction process to improve the yield and quality of crude oils.

## Figures and Tables

**Table 1:** Oil yield/recovery from Baltic herring whole fish and by-products from solvent (reference) and enzymatic extractions. The oil yields (g/100 g fw) for pre-tests are presented as mean ± standard deviation and oil recovery as percentage of the solvent-extracted oil. Actual test samples are presented as g/100 g fw and percentage of the solvent-extracted oil.

	Solvent Extraction g	Alcalase		Neutrase		Protamex	
		35 min g/%	70 min g/%	35 min g/%	70 min g/%	35 min g/%	70 min g/%
Pre-test (whole fish)	4.89 ± 1.28	2.24 ± 0.56/45.8	-	2.11 ± 0.25/43.1	-	2.47 ± 0.71/50.5	-
Whole fish *	9.00 ± 0.46	4.09/45.4	3.85/42.8	5.39/59.9	6.25/69.4	4.52/50.2	6.08/67.6
By-products *	9.46 ± 0.23	4.57/48.3	5.13/54.2	5.17/54.7	5.44/57.5	4.57/48.3	6.14/64.9

* In the actual test, the oil yield and recovery were determined after pooling of the triplicates of each enzymatic treatment to take into consideration the differences among different batches and to limit the total number of analyses.

**Table 2:** Fatty acid composition of enzymatically extracted oils and solvent-extracted oil (reference) from whole Baltic herring. The values are presented as mass percentages of the total fatty acid content. The results are shown as mean value ± standard deviation of the three analytical replicates.

	2-Way ANOVA			Solvent Extraction	Alcalase		Neutrase		Protamex	
Fatty Acid	Enzyme	Time	E × T		35 min	70 min	35 min	70 min	35 min	70 min
14:0	**	n	**	6.56 ± 0.17 ^c,d^	6.74 ± 0.06 ^d^	6.18 ± 0.05 ^b^	6.55 ± 0.10 ^c,d^	6.31 ± 0.08 ^b,c^	5.76 ± 0.11 ^a^	6.52 ± 0.12 ^c,d^
14:1(n-5)	**	**	**	0.15 ± 0.01 ^a^	0.72 ± 0.00 ^e^	0.71 ± 0.00 ^e^	0.83 ± 0.02 ^f^	0.65 ± 0.01 ^d^	0.49 ± 0.01 ^b^	0.55 ± 0.04 ^c^
16:0	**	n	n	17.77 ± 0.10 ^a^	17.62 ± 0.15 ^a^	17.81 ± 0.11 ^a^	17.92 ± 0.06 ^a,b^	18.20 ± 0.22 ^c^	18.43 ± 0.27 ^c^	18.30 ± 0.29 ^b,c^
16:1(n-7)	**	n	**	5.27 ± 0.12 ^c^	4.93 ± 0.03 ^b^	4.61 ± 0.05 ^a^	4.60 ± 0.03 ^a^	4.61 ± 0.06 ^a^	4.73 ± 0.07 ^a,b^	4.89 ± 0.05 ^b^
17:1(n-8)	n	n	*	0.31 ± 0.01 ^a,b^	0.29 ± 0.02 ^a^	0.40 ± 0.03 ^c^	0.33 ± 0.01 ^a,b^	0.32 ± 0.01 ^a,b^	0.36 ± 0.01 ^b,c^	0.33 ± 0.07 ^a.b^
18:0	**	**	**	1.24 ± 0.02 ^c,d^	1.15 ± 0.01 ^a^	1.31 ± 0.01 ^e^	1.27 ± 0.00 ^d^	1.21 ± 0.02 ^b,c^	1.39 ± 0.01 ^f^	1.19 ± 0.02 ^b^
18:1(n-9)t	n	n	*	0.01 ± 0.02 ^a^	0.05 ± 0.02 ^b,c^	0.07 ± 0.00 ^c^	0.04 ± 0.01 ^a.b,c^	0.05 ± 0.02 ^b,c^	0.07 ± 0.01 ^c^	0.03 ± 0.00 ^a,b^
18:1(n-9)c	n	*	**	25.22 ± 0.10 ^d^	25.22 ± 0.11 ^c,d^	23.55 ± 0.08 ^a^	23.31 ± 0.05 ^a^	24.64 ± 0.22 ^b,c^	23.70 ± 0.31 ^a^	24.79 ± 0.21 ^b^
18:1(n-7)	n	n	*	2.66 ± 0.02 ^c^	2.51 ± 0.00 ^a^	2.55 ± 0.02 ^a,b^	2.58 ± 0.01 ^a,b^	2.51 ± 0.03 ^a^	2.63 ± 0.01 ^b,c^	2.54 ± 0.08 ^a^
18:2(n-6)t	n	n	n	0.08 ± 0.01 ^a^	0.41 ± 0.02 ^b,c^	0.36 ± 0.02 ^b,c^	0.46 ± 0.12 ^c^	0.42 ± 0.03 ^b,c^	0.34 ± 0.01 ^b^	0.40 ± 0.01 ^b,c^
18:2(n-6)c	n	n	**	4.29 ± 0.03 ^a^	4.62 ± 0.04 ^b^	4.94 ± 0.03 ^c^	4.70 ± 0.03 ^b^	5.00 ± 0.05 ^c^	5.09 ± 0.12 ^c^	4.58 ± 0.12 ^b^
18:3(n-6)	n	n	*	0.12 ± 0.00 ^a^	0.15 ± 0.01 ^a,b^	0.17 ± 0.00 ^b^	0.17 ± 0.01 ^b^	0.12 ± 0.04 ^a^	0.16 ± 0.01 ^a,b^	0.16 ± 0.00 ^a,b^
18:3(n-3)	n	n	n	2.39 ± 0.03 ^a,b^	2.48 ± 0.03 ^a,b^	2.97 ± 0.04 ^b^	2.83 ± 0.01 ^a,b^	2.63 ± 0.02 ^a^	2.96 ± 0.08 ^b^	2.55 ± 0.03 ^a,b^
18:4(n-3)	*	*	**	2.32 ± 0.01 ^a^	2.59 ± 0.03 ^b,c^	2.77 ± 0.01 ^d^	2.74 ± 0.02 ^d^	2.48 ± 0.03 ^b^	2.81 ± 0.08 ^d^	2.62 ± 0.08 ^c^
20:0	n	n	n	0.16 ± 0.01	0.13 ± 0.01	0.17 ± 0.02	0.17 ± 0.03	0.14 ± 0.04	0.16 ± 0.02	0.15 ± 0.03
20:1(n-9)	**	**	**	2.22 ± 0.02 ^c^	2.34 ± 0.01 ^d^	2.08 ± 0.01 ^b^	2.23 ± 0.02 ^c^	2.34 ± 0.01 ^d^	1.94 ± 0.04 ^a^	2.36 ± 0.02 ^d^
20:1(n-11)	*	*	**	0.31 ± 0.00 ^b^	0.35 ± 0.01 ^c,d^	0.30 ± 0.01 ^b^	0.33 ± 0.02 ^b,c^	0.35 ± 0.00 ^d^	0.28 ± 0.01 ^a^	0.36 ± 0.01 ^d^
20:2(n-6)	**	**	**	1.52 ± 0.02 ^a^	1.78 ± 0.02 ^d^	1.69 ± 0.02 ^c^	1.71 ± 0.01 ^c^	1.88 ± 0.02 ^e^	1.61 ± 0.03 ^b^	1.82 ± 0.04 ^d^
20:3(n-6)	*	n	n	0.00 ± 0.00 ^a^	0.04 ± 0.02 ^b^	0.05 ± 0.02 ^b^	0.08 ± 0.01^.c^	0.05 ± 0.02 ^b^	0.04 ± 0.00 ^b^	0.04 ± 0.00 ^b^
20:4(n-6)	*	n	**	0.46 ± 0.01 ^c,d^	0.38 ± 0.03 ^a^	0.45 ± 0.01 ^c,d^	0.43 ± 0.01 ^b,c^	0.39 ± 0.01 ^a^	0.48 ± 0.02 ^d^	0.40 ± 0.02 ^a.b^
20:3(n-3)	**	**	**	1.07 ± 0.02 ^a^	1.26 ± 0.02 ^c^	1.16 ± 0.01 ^b^	1.20 ± 0.01 ^b^	1.31 ± 0.02 ^d^	1.10 ± 0.02 ^a^	1.31 ± 0.03 ^c,d^
20:4(n-3)	**	*	**	1.50 ± 0.03 ^a^	1.75 ± 0.02 ^c^	1.59 ± 0.01 ^b^	1.73 ± 0.01 ^c^	1.77 ± 0.03 ^c^	1.53 ± 0.03 ^a,b^	1.78 ± 0.05 ^c^
20:5(n-3)	*	**	**	6.46 ± 0.08 ^b^	6.11 ± 0.06 ^a^	6.93 ± 0.03 ^c^	6.74 ± 0.03 ^b,c^	6.06 ± 0.09 ^a^	7.33 ± 0.20 ^d^	6.06 ± 0.16 ^a^
22:0	n	*	*	0.08 ± 0.00 ^a,b^	0.09 ± 0.01 ^a,b^	0.09 ± 0.01 ^a,b,c^	0.09 ± 0.01 ^b,c^	0.07 ± 0.02 ^a,b^	0.11 ± 0.01 ^c^	0.07 ± 0.01 ^a^
22:1(n-9)	*	n	**	0.41 ± 0.01 ^a^	0.49 ± 0.01 ^c^	0.45 ± 0.00 ^b^	0.49 ± 0.01 ^c^	0.49 ± 0.01 ^c^	0.42 ± 0.03 ^a^	0.48 ± 0.02 ^c^
22:1(n-11)	n	n	n	0.06 ± 0.01	0.08 ± 0.01	0.08 ± 0.00	0.08 ± 0.01	0.06 ± 0.00	0.07 ± 0.01	0.08 ± 0.03
22:2(n-6)	n	*	*	0.63 ± 0.03 ^a^	0.86 ± 0.02 ^c,d^	0.80 ± 0.01 ^b,c^	0.84 ± 0.02 ^c,d^	0.91 ± 0.10 ^d^	0.70 ± 0.02 ^a,b^	0.91 ± 0.03 ^d^
22:3(n-3)	n	n	n	0.20 ± 0.02	0.22 ± 0.03	0.24± 0.01	0.22 ± 0.03	0.19 ± 0.01	0.24 ± 0.01	0.21 ± 0.03
22:4(n-6)	*	*	*	0.69 ± 0.01 ^a,b^	0.76 ± 0.02 ^b,c,d^	0.75 ± 0.03 ^b,c^	0.76 ± 0.02 ^b.c,d^	0.84 ± 0.02 ^d^	0.65 ± 0.08 ^a^	0.81 ± 0.04 ^c,d^
22:5(n-3)	n	n	n	0.88 ± 0.02	0.89 ± 0.01	0.91 ± 0.01	0.91 ± 0.01	0.91 ± 0.02	0.88 ± 0.01	0.92 ± 0.05
22:6(n-3)	*	*	**	11.50 ± 0.11 ^d^	9.68 ± 0.10 ^a^	10.73 ± 0.05 ^c^	10.23 ± 0.04 ^b^	9.45 ± 0.15 ^a^	10.76 ± 0.29 ^c^	9.43 ± 0.27 ^a^
24:1(n-9)	n	n	n	1.51 ± 0.32	1.08 ± 0.06	1.12 ± 0.05	1.06 ± 0.07	1.21 ± 0.13	1.13 ± 0.08	1.11 ± 0.17
24:4(n-3)	**	**	**	1.38 ± 0.17 ^a,b^	1.60 ± 0.03 ^b,c,d^	1.47 ± 0.07 ^a,b,c^	1.70 ± 0.02 ^c,d^	1.75 ± 0.04 ^d^	1.20 ± 0.02 ^a^	1.61 ± 0.04 ^b,c,d^
24:5(n-3)	**	n	*	0.54 ± 0.02 ^a,b^	0.63 ± 0.07 ^b,c^	0.54 ± 0.03 ^a,b^	0.67 ± 0.04 ^c^	0.68 ± 0.05 ^c^	0.46 ± 0.02 ^a^	0.61 ± 0.04 ^b,c^
SFA	*	n	n	25.81 ± 0.26	25.72 ± 0.24	25.56 ± 0.17	26.02 ± 0.07	25.93 ± 0.32	25.84 ± 0.39	26.23 ± 0.44
MUFA	n	*	**	38.14 ± 0.28 ^b^	38.06 ± 0.18 ^b^	35.92 ± 0.10 ^a^	35.86 ± 0.03 ^a^	37.25 ± 0.29 ^b^	35.83 ± 0.53 ^a^	37.56 ± 0.44 ^b^
PUFA	n	n	**	36.04 ± 0.29 ^a^	36.2 ± 0.40 ^a^	38.52 ± 0.27 ^b^	38.13 ± 0.07 ^b^	36.83 ± 0.56 ^a^	38.33 ± 0.91 ^b^	36.21 ± 0.89 ^b^
n-3	n	*	**	28.25 ± 0.25 ^b,c^	27.21 ± 0.30 ^a,b^	29.31 ± 0.17 ^c^	28.97 ± 0.07 ^c^	27.23 ± 0.38 ^a^	29.27 ± 0.71 ^c^	27.09 ± 0.69 ^a,b^
n-6	*	*	n	7.79 ± 0.05 ^a^	9.01 ± 0.10 ^b^	9.21 ± 0.10 ^b^	9.16 ± 0.12 ^b^	9.60 ± 0.21 ^c^	9.06 ± 0.22 ^b^	9.12 ± 0.20 ^b^

Different letters indicate significant differences (*p* < 0.05) detected by one-way ANOVA and Tukey HSD. * *p* < 0.05, ** *p* < 0.001, n not significant as determined by statistical comparisons with two-way ANOVA.

**Table 3:** Fatty acid composition of enzymatically extracted oils and solvent-extracted oil (reference) from Baltic herring by-products. The values are presented as mass percentages of the total fatty acid content. The results are shown as mean value ± standard deviation of the three analytical replicates.

	2-Way ANOVA			Solvent Extraction	Alcalase		Neutrase		Protamex	
Fatty Acid	Enzyme	Time	E × T		35 min	70 min	35 min	70 min	35 min	70 min
14:0	n	n	n	6.69 ± 0.09	6.53 ± 0.03	6.82 ± 0.86	6.29 ± 0.13	6.94 ± 0.11	6.55 ± 0.04	6.58 ± 0.02
14:1(n-5)	*	*	**	0.17 ± 0.01 ^a^	0.61 ± 0.04 ^c,d^	0.56 ± 0.09 ^c^	0.41 ± 0.05 ^b^	0.68 ± 0.03 ^d^	0.68 ± 0.03 ^d^	0.69 ± 0.02 ^d^
16:0	n	n	n	18.39 ± 0.09	17.95 ± 0.06	19.35 ± 2.37	18.32 ± 0.13	18.71 ± 0.50	17.79 ± 0.04	17.76 ± 0.01
16:1(n-7)	**	**	**	4.79 ± 0.06 ^b^	5.06 ± 0.01 ^c^	5.38 ± 0.08 ^d^	4.55 ± 0.01 ^a^	4.88 ± 0.02 ^b^	4.82 ± 0.01 ^b^	4.89 ± 0.01 ^b^
17:1(n-8)	n	n	n	0.29 ± 0.02 ^a^	0.35 ± 0.03 ^a,b^	0.36 ± 0.03 ^b^	0.30 ± 0.05 ^a,b^	0.34 ± 0.01 ^a,b^	0.30 ± 0.01 ^a,b^	0.34 ± 0.04 ^a,b^
18:0	n	n	n	1.18 ± 0.01 ^a^	1.35 ± 0.01 ^a,b^	1.39 ± 0.15 ^a.b^	1.21 ± 0.00 ^a^	1.46 ± 0.22 ^b^	1.27 ± 0.00 ^a,b^	1.21 ± 0.00 ^a^
18:1(n-9)t	n	n	*	0.00 ± 0.00 ^a^	0.06 ± 0.02 ^b,c^	0.05 ± 0.01 ^b^	0.04 ± 0.02 ^a.b^	0.09 ± 0.03 ^c^	0.06 ± 0.01 ^b,c^	0.05 ± 0.01 ^b,c^
18:1(n-9)c	n	n	n	24.97 ± 0.21	25.10 ± 0.05	25.51 ± 1.09	25.25 ± 0.11	24.98 ± 0.17	24.83 ± 0.01	24.65 ± 0.04
18:1(n-7)	*	n	n	2.56 ± 0.02 ^a^	2.60 ± 0.01 ^a,b^	2.68 ± 0.12 ^b^	2.55 ± 0.02 ^a^	2.60 ± 0.03 ^a,b^	2.55 ± 0.00 ^a^	2.55 ± 0.01 ^a^
18:2(n-6)t	n	n	n	0.12 ± 0.01 ^a^	0.41 ± 0.01 ^b^	0.42 ± 0.03 ^b^	0.42 ± 0.01 ^b^	0.52 ± 0.07 ^c^	0.42 ± 0.05 ^b^	0.41 ± 0.01 ^b^
18:2(n-6)c	n	n	n	4.53 ± 0.04	4.69 ± 0.01	4.40 ± 0.63	4.81 ± 0.05	4.56 ± 0.05	4.78 ± 0.01	4.80 ± 0.01
18:3(n-6)	n	n	n	0.11 ± 0.00 ^a^	0.15 ± 0.00 ^b^	0.16 ± 0.02 ^b^	0.15 ± 0.00 ^b^	0.15 ± 0.02 ^b^	0.17 ± 0.01 ^b^	0.16 ± 0.01 ^b^
18:3(n-3)	n	n	n	2.35 ± 0.06 ^a,b^	2.53 ± 0.05 ^b^	2.19 ± 0.32 ^a^	2.39 ± 0.03 ^a,b^	2.35 ± 0.07 ^a,b^	2.42 ± 0.02 ^a,b^	2.50 ± 0.07 ^b^
18:4(n-3)	n	n	n	2.10 ± 0.07 ^a^	2.42 ± 0.01 ^b^	2.26 ± 0.29 ^a,b^	2.42 ± 0.02 ^b^	2.34 ± 0.03 ^a,b^	2.51 ± 0.00 ^b^	2.47 ± 0.01 ^b^
20:0	n	n	n	0.15 ± 0.01	0.16 ± 0.01	0.17 ± 0.02	0.15 ± 0.04	0.16 ± 0.02	0.15 ± 0.01	0.14 ± 0.01
20:1(n-9)	*	n	*	2.53 ± 0.08 ^a,b^	2.40 ± 0.01 ^a^	2.49 ± 0.11 ^a,b^	2.59 ± 0.01 ^b^	2.50 ± 0.01 ^a,b^	2.42 ± 0.00 ^a^	2.45 ± 0.01 ^a,b^
20:1(n-11)	n	n	*	0.38 ± 0.01 ^a,b^	0.37 ± 0.01 ^a^	0.39 ± 0.02 ^a,b^	0.40 ± 0.00 ^b^	0.39 ± 0.01 ^a,b^	0.38 ± 0.02 ^a,b^	0.40 ± 0.00 ^a,b^
20:2(n-6)	*	n	n	1.94 ± 0.02 ^b,c^	1.77 ± 0.02 ^a,b^	1.72 ± 0.23 ^a^	2.00 ± 0.04 ^c^	1.85 ± 0.05 ^a,b,c^	1.86 ± 0.01 ^a,b,c^	1.94 ± 0.02 ^b,c^
20:3(n-6)	n	n	n	0.00 ± 0.00 ^a^	0.04 ± 0.02 ^b^	0.05 ± 0.01 ^b^	0.04 ± 0.01^.b^	0.02 ± 0.01 ^a,b^	0.05 ± 0.01 ^b^	0.04 ± 0.01 ^b^
20:4(n-6)	n	n	n	0.38 ± 0.01 ^a,b^	0.38 ± 0.02 ^a,b^	0.33 ± 0.04 ^a,b^	0.34 ± 0.01 ^a,b^	0.33 ± 0.03 ^a^	0.39 ± 0.05 ^b^	0.37 ± 0.01 ^a,b^
20:3(n-3)	*	n	n	1.43 ± 0.02 ^c,d^	1.25 ± 0.01 ^a,b^	1.18 ± 0.16 ^a^	1.46 ± 0.02 ^d^	1.30 ± 0.03 ^a,b,c^	1.31 ± 0.03 ^a,b,c^	1.37 ± 0.01 ^b,c,d^
20:4(n-3)	n	n	n	1.66 ± 0.02 ^a^	1.77 ± 0.03 ^a,b^	1.67 ± 0.25 ^a^	1.92 ± 0.03 ^b^	1.80 ± 0.05 ^a,b^	1.81 ± 0.02 ^a,b^	1.85 ± 0.00 ^a,b^
20:5(n-3)	n	n	n	5.62 ± 0.18	5.83 ± 0.01	5.36 ± 0.76	5.53 ± 0.06	5.35 ± 0.10	5.87 ± 0.01	5.88 ± 0.01
22:0	n	n	n	0.07 ± 0.01 ^a,b^	0.08 ± 0.01 ^a,b^	0.09 ± 0.02 ^b^	0.08 ± 0.01 ^a,b^	0.06 ± 0.01 ^a^	0.08 ± 0.00 ^a,b^	0.08 ± 0.02 ^a,b^
22:1(n-9)	*	n	*	0.52 ± 0.02	0.51 ± 0.00	0.53 ± 0.01	0.55 ± 0.00	0.53 ± 0.01	0.53 ± 0.02	0.52 ± 0.01
22:1(n-11)	n	n	n	0.08 ± 0.01	0.09 ± 0.01	0.09 ± 0.01	0.09 ± 0.00	0.08 ± 0.01	0.08 ± 0.00	0.08 ± 0.02
22:2(n-6)	n	n	n	1.00 ± 0.02 ^a,b^	0.92 ± 0.00 ^a^	0.94 ± 0.12 ^a,b^	1.05 ± 0.03 ^b^	0.96 ± 0.04 ^a,b^	0.97 ± 0.04 ^a,b^	1.01 ± 0.02 ^a,b^
22:3(n-3)	n	n	n	0.21 ± 0.02	0.22 ± 0.01	0.23 ± 0.09	0.19 ± 0.02	0.22 ± 0.03	0.20 ± 0.03	0.20 ± 0.01
22:4(n-6)	*	n	n	0.99 ± 0.02 ^c^	0.78 ± 0.03 ^a^	0.78 ± 0.12 ^a^	0.94 ± 0.01 ^b.c^	0.81 ± 0.08 ^a^	0.83 ± 0.02 ^a.b^	0.88 ± 0.01 ^a,b,c^
22:5(n-3)	n	n	n	0.95 ± 0.01	0.92 ± 0.01	0.78 ± 0.12	0.96 ± 0.02	0.88 ± 0.02	0.93 ± 0.02	0.95 ± 0.02
22:6(n-3)	n	n	n	9.26 ± 0.34	9.18 ± 0.05	8.27 ± 1.39	8.76 ± 0.09	8.48 ± 0.13	9.28 ± 0.03	9.03 ± 0.03
24:1(n-9)	n	n	*	1.70 ± 0.43	1.18 ± 0.04	1.10 ± 0.01	1.07 ± 0.01	1.20 ± 0.02	1.14 ± 0.07	1.09 ± 0.07
24:4(n-3)	*	n	n	2.09 ± 0.32	1.67 ± 0.04	1.64 ± 0.19	2.01 ± 0.03	1.82 ± 0.09	1.80 ± 0.03	1.91 ± 0.05
24:5(n-3)	*	n	n	0.79 ± 0.04 ^b^	0.65 ± 0.04 ^a,b^	0.63 ± 0.09 ^a^	0.78 ± 0.07 ^b^	0.66 ± 0.03 ^a,b^	0.77 ± 0.06 ^a,b^	0.75 ± 0.08 ^a,b^
SFA	n	n	n	26.48 ± 0.17	26.06 ± 0.06	27.81 ± 3.41	26.05 ± 0.26	27.34 ± 0.67	25.84 ± 0.08	25.77 ± 0.04
MUFA	*	n	n	38.00 ± 0.66	38.35 ± 0.04	39.15 ± 1.29	37.79 ± 0.10	38.26 ± 0.22	37.79 ± 0.06	37.72 ± 0.10
PUFA	n	n	n	35.52 ± 0.75	35.59 ± 0.02	33.04 ± 4.69	36.16 ± 0.24	34.40 ± 0.72	36.37 ± 0.05	36.51 ± 0.11
n-3	n	n	n	26.45 ± 0.74	26.43 ± 0.04	24.25 ± 3.56	26.41 ± 0.30	25.20 ± 0.48	26.90 ± 0.06	26.89 ± 0.11
n-6	n	n	n	9.07 ± 0.05 ^a,b^	9.16 ± 0.03 ^a,b^	8.78 ± 1.14 ^a^	9.75 ± 0.05 ^b^	9.21 ± 0.25 ^a,b^	9.47 ± 0.01 ^a,b^	9.62 ± 0.03 ^a,b^

Different letters indicate significant differences (*p* < 0.05) detected by one-way ANOVA and Tukey HSD. * *p* < 0.05, ** *p* < 0.001, n not significant as determined by statistical comparisons with two-way ANOVA.

**Table 4:** *p*-Anisidine values (AV), peroxide values (PV) and total oxidation product (TOTOX) values of different enzymatically extracted oils. The results for AV and PV are shown as mean value ± standard deviation. Significant differences (*p* < 0.05) were determined by one-way ANOVA and Tukey HSD.

	Alcalase		Neutrase		Protamex	
	35 min	70 min	35 min	70 min	35 min	70 min
**Whole fish**						
AV	11.10 ± 0.61 ^a^	16.87 ± 2.77 ^b,c^	16.91 ± 0.78 ^b,c^	15.25 ± 0.03 ^a,b^	21.08 ± 1.31 ^c,d^	25.84 ± 0.54 ^d^
PV	22.95 ± 1.92 ^a^	53.66 ± 6.90 ^b^	54.44 ± 7.27 ^b^	43.65 ± 2.89 ^b^	57.58 ± 6.50 ^b^	75.65 ± 7.40 ^c^
TOTOX	54.88	124.19	125.80	102.56	136.23	177.14
**By-products**						
AV	16.34 ± 0.37 ^b,c^	25.88 ± 5.80 ^c^	5.60 ± 1.14 ^a^	13.35 ± 0.29 ^a,b^	12.05 ± 0.30 ^a,b^	9.94 ± 0.03 ^a,b^
PV	49.67 ± 1.53 ^c^	68.43 ± 1.01 ^d^	23.56 ± 0.19 ^a^	52.05 ± 2.00 ^c^	35.21 ± 0.68 ^b^	36.77 ± 0.64 ^b^
TOTOX	115.69	162.74	52.72	117.44	82.46	83.48

Different letters indicate significant differences (*p* < 0.05).

**Table 5:** All identified volatile compounds of enzymatically extracted Baltic herring oil. The compounds were identified based on the literature, NIST library and external standards (STD). Retention indices for HS-SPME-GC-MS of some volatile compounds are also presented. The column was SPB^®^-624 Fused Silica Capillary Column 60 m × 0.25 mm × 1.4 µm.

Compound Number	Compound	Identification Method	RT (min)	RI ^1^
1	Acetaldehyde	STD	6.51	
2	2-Propenal	MS	9.79	
3	Propanal	STD	9.97	
4	Butanal	STD	14.55	638
5	2,3-Butanedione	MS	14.64	640
6	Unknown 1	-	14.84	644
7	Formic acid	MS	15.96	667
8	1,3-Butanediol	MS	14.64	676
9	Acetic acid	STD	17.44	696
10	3-Methylbutanal	MS	17.67	700
11	Heptane	STD	17.84	704
12	2-Methylbutanal	MS	18.05	709
13	(*Z*)-2-Butenal	MS	18.13	711
14	2-Ethylfuran	STD	18.85	727
15	1-Penten-3-one	MS	19.31	738
16	1-Penten-3-ol	STD	19.44	741
17	2,3-Pentanedione	MS	19.68	746
18	Cyclopentanol	MS	19.73	747
19	Propanoic acid	MS	21.67	787
20	(*E*)-2-Pentenal	STD	22.41	801
21	Hexanal	STD	24.44	851
22	2-Methyl-4-pentenal	MS	24.48	852
23	3-Hexanol	MS	25.71	880
24	2,4-Hexadien-1-ol	MS	26.84	905
25	(*E*)-2-Hexenal	STD	27.37	920
26	Heptanal	STD	28.69	954
27	(*E*,*E*)-2,4-Hexadienal	STD	30.05	988
28	Unknown 2	-	30.58	1002
29	2-Pentylfuran	STD	31.23	1020
30	(*Z*)-2-Heptenal	MS	31.48	1027
31	(*E*)-2-(2-Pentenyl)furan	MS	31.72	1034
32	Octanal	STD	32.55	1058
33	Hexanoic acid	MS	32.62	1060
34	(*E*,*Z*)-2,4-Heptadienal	MS	33.22	1076
35	(*E*,*E*)-2,4-Heptadienal	MS	33.81	1091
36	(*E*,*E*)-3,5-Octadien-2-one	MS	35.57	1146
37	2-Nonanone	MS	35.73	1151
38	Nonanal	STD	36.08	1161
39	5-Ethyl-2(5H)-furanone	MS	36.45	1173
40	(*E*,*E*)-3,5-Octadien-2-one	MS	36.52	1175
41	Nonanoic acid	MS	41.94	1334

^1^ The retention indices are calculated in regard to a series of *n*-alkanes.

## Data Availability

Not applicable.
